# Do stretch sensors expressed by aortic baroreceptors interact with circulating estradiol to mediate baroreflex sensitivity in hypertension?

**DOI:** 10.3389/fnins.2026.1848764

**Published:** 2026-06-10

**Authors:** Saleh Salman, Gavyn Navarro, Khalid Elsaafien

**Affiliations:** 1 Department of Molecular and Cellular Physiology, LSU Health Shreveport, Shreveport, LA, United States; 2 Center for Cardiovascular Diseases and Sciences, LSU Health Shreveport, Shreveport, LA, United States

**Keywords:** aortic baroreceptors, baroreflex, blood pressure, estradiol, hypertension, nodose ganglia, sensory vagal afferents, stretch sensors

## Abstract

Hypertension, or high blood pressure, is a major risk factor for cardiovascular disease, the leading cause of mortality worldwide. The incidence and the severity of hypertension is higher in middle-aged men than women. The hallmark of hypertension is an increased sympathetic nerve activity to the cardiovascular organs. One mechanism that regulates sympathetic nerve activity is the homeostatic baroreflex which maintains blood pressure at optimal levels for survival. Baroreceptive nerve endings innervating the aortic arch detect stretch at the vascular wall and convey these signals to the hindbrain which subsequently modulates sympathetic nerve activity. Although the baroreflex was described more than 80 years ago, the specific molecular, structural, and functional phenotype of aortic baroreceptors remain to be fully elucidated. Several recent studies suggest the involvement of various ion channels, termed as “Stretch Sensors”, in detecting vascular stretch. Stretch sensors are diverse, and they include *Piezo*, transient receptor potential, acid sensing ion, and epithelial sodium channels. Thus, stretch sensors engaged by aortic baroreceptors may evoke baroreception, leading to the regulation of sympathetic nerve activity and blood pressure. In pathophysiological conditions, impaired engagement of stretch sensors may lead to sympathetic nerve overactivity and sustained elevations in blood pressure. Furthermore, ovarian hormones, particularly estradiol, may interact with stretch sensors, increasing baroreflex sensitivity and leading to cardioprotective effects in women. However, low circulating levels of estradiol, such as in post-menopause, can lead to reduced baroreflex sensitivity, hypertension and cardiovascular disease. In this review, we discuss stretch sensors expressed by aortic baroreceptors, the role they play in baroreception and blood pressure regulation, interplay with estradiol, and the role they play the development of hypertension and mediating sex-specific differences.

## 1 Introduction

Hypertension or high blood pressure (BP) is a risk factor for cardiovascular disease, the leading cause of death worldwide (Members et al., 2025). High BP affects men and women differently. Although the incidence is higher amongst middle-aged men, the prevalence significantly increases to similar rates to men in older-women (Fryar et al., 2024). Despite the plethora of anti-hypertensive treatments, approximately 30% of hypertensive patients suffer from drug-resistant hypertension, where BP is uncontrollable despite the use of a combination of medications (Carey et al., 2019; Members et al., 2025). Augmented sympathetic nerve activity (SNA) is well-documented in patients with resistant hypertension (Smith et al., 2002, 2004). However, the mechanisms underlying these chronic elevations are not well understood. One mechanism that influences SNA is the arterial baroreflex (Kirchheim, 1976; Brown, 1980). Several studies suggest that impairment or reduced baroreflex sensitivity is linked to sympathetic overactivity and hypertension (Rodrigues et al., 2011; Oliveira-Sales et al., 2014, 2016). Therefore, investigating the role the baroreflex plays in regulating BP and the pathogenesis of hypertension is crucial for identifying novel therapeutic targets.

The homeostatic baroreflex monitors arterial stretch to exert moment-to-moment reflex regulation of BP at levels optimal for survival (Kirchheim, 1976; Brown, 1980). A subset of sensory vagal afferents employs stretch sensors that innervate the aortic arch and the carotid sinus to sense and monitor stretch at the arterial wall and convey these signals to the hindbrain (Benarroch, 2008; Wehrwein and Joyner, 2013; Elsaafien et al., 2022; Scott et al., 2025). An increase in BP exerts stretch at the vascular wall of the aortic arch and carotid sinus, activating stretch sensitive nerve endings, termed as baroreceptors (Kirchheim, 1976; Baumer-Harrison et al., 2024). This activation is conveyed through the vagus nerve (cranial nerve X) to the nucleus of the solitary tract (NTS) (Elsaafien et al., 2022; Scott et al., 2025). Subsequently, SNA is reduced and parasympathetic nerve activity is increased, restoring BP and heart rate (HR) to homeostatic levels (Spyer, 1989; Andresen and Kunze, 1994). This mechanism and reflex allow for the moment-to-moment feedback monitoring which control and regulate BP and HR.

Stretch sensitive reflexes regulating BP were first observed by Astley Cooper in 1836 (Heymans, 1960). A series of follow-up studies done between 1900s−1930s led to characterizing these reflexes as the moment-to-moment feedback mechanisms regulating BP and HR, which we know today as the baroreflex (Siciliano, 1900; Hering, 1923, 1925; Pagano, 1980). Subsequently, between 1950s−1990s researchers begun investigating the role the baroreflex plays in the development of hypertension (Krieger and Marseillan, 1963). At that time, necessity approaches were the gold-standard of research, where the question probed was whether a pathway is necessary for the pathogenesis of a disease. If it is, then ablating that specific mechanism would lead to the development of the disease. This gave birth to the sino-aortic denervation (SAD) model, where sensory nerve endings innervating the aortic arch and the carotid sinus are ablated (Krieger and Marseillan 1963; Di Rienzo et al., 1991). Findings from these studies would suggest that the baroreflex is not involved in the pathogenesis of hypertension, as ablating “baroreceptor-sensitive nerve endings” did not lead to the development of hypertension (AW, 1980; Norman et al., 1981; Schreihofer and Sved, 1992). These studies lead to the consensus in the field that arterial baroreceptors are only involved in the moment-to-moment regulation of BP and that they are not implicated in the development of hypertension. These conclusions and interpretations almost put the field of arterial baroreceptors into a stand-still by the early 2000s. However, with recent advancement in selective approaches and technologies in biomedical research, over the past decade many investigators have begun revisiting arterial baroreceptors. Approaches such as the Cre-LoxP system of genetic tailoring, virally directed neuroanatomical tract tracing, and multi-omics revealed that arterial baroreceptors utilize the mechanically gated ion channels *Piezo1 & 2* in sensing stretch (Zeng et al., 2018; Min et al., 2019; Elsaafien et al., 2022; Cheng et al., 2025). Subsequently, a recent study utilized *in vivo* two-photon calcium imaging to assess whether arterial baroreceptors sense stretch differently during physiological and hypertensive conditions. It was demonstrated that arterial baroreceptor's abilities to sense stretch are impaired in a mouse model of hypertension (Baumer-Harrison et al., 2024). Ultimately, leading to several preclinical and clinical studies demonstrating that baroreceptor activation therapy may be beneficial in hypertension (Heusser et al., 2010; Scheffers et al., 2010; Heusser et al., 2016). Overall, advanced approaches and technologies have allowed us to begin to unravel the molecular, structural, and functional phenotype of arterial baroreceptors in health and disease state. This understanding is crucial to identify novel therapeutic targets that alleviate hypertension.

The present review discusses recent advancement and identifies current gaps in knowledge in the field of studying arterial baroreceptors and the baroreflex. We will highlight and address the following fundamental questions; 1) are baroreceptors and the baroreflex involved in the development of hypertension? 2) how do baroreceptors sense stretch under physiological and pathological conditions? 3) are *Piezo* channels the exclusive stretch sensors employed by arterial baroreceptors? 4) do ovarian hormones interact with arterial baroreceptors to mediate sex-specific differences in hypertension?

## 2 Arterial baroreceptors and the development of a hypertensive phenotype

The role arterial baroreceptors and the baroreflex play in the development of hypertension is an evolving area of research. Early studies relied on the sino-aortic denervation (SAD) model to evaluate whether arterial baroreceptors are necessary for the development of hypertension (Krieger and Marseillan, 1963; Krieger, 1964; Masson et al., 1966; Krieger, 1967). The SAD model was pioneered by Eduardo Krieger, where all sensory nerve endings innervating the aortic arch and the carotid sinus are ablated (Krieger and Marseillan, 1963; Krieger, 1964, 1967; Sapru and Krieger, 1981; Krieger, 1988; Krieger et al., 2022). This involves sectioning the aortic depressor nerve and the superior laryngeal nerve just distal to their junction with the vagus. Krieger et al. found that rats subjected to SAD developed hypertension (Krieger and Marseillan, 1963; Krieger, 1964, 1967), which was later replicated in cats (Di Rienzo et al., 1991), rabbits (Ramchandra et al., 2003), and mice (Fazan Jr et al., 2005; Rodrigues et al., 2011). These results support the notion that impairing arterial baroreceptors may promote the development of hypertension. However, follow-up studies did not observe a hypertensive phenotype after SAD (AW, 1980). Instead, these studies found that rats subjected to SAD were normotensive but exhibited increased arterial pressure lability (Norman et al., 1981; Buchholz et al., 1986; Barron et al., 1989; Schreihofer and Sved, 1992). One interpretation of these results is that arterial baroreceptors are not implicated in the development of hypertension, but rather, only contribute to the short-term regulation of blood pressure. However, inherent variability associated with the SAD model likely contributed to discrepant results. That is, SAD strips away nerve endings innervating the carotid sinus, which likely ablates sensory endings that function as arterial chemoreceptors (Porzionato et al., 2019). Indeed, a recent study demonstrated that animals undergoing SAD do not respond to challenging arterial chemoreceptors (Silva et al., 2015). Opposite of what was expected with the inhibition of the baroreflex, ablating the chemoreflex lowers sympathetic activity and promotes vasodilation (Seagard et al., 1990; Silva et al., 2015; Porzionato et al., 2019). This presents the possibility that SAD may inadvertently lesion the arterial chemoreceptors and negate the sympatho-excitation expected with baroreceptor ablation, and consequently, produce a normotensive phenotype. Thus, an approach to selectively target arterial baroreceptors and distinguish them from arterial chemoreceptors is warranted. Such an approach will allow us to begin investigating arterial baroreceptors and the role they play in the development of hypertension.

## 3 Aortic baroreceptors

Over the past decade, several studies have attempted to selectively label arterial baroreceptors and delineate them from arterial chemoreceptors. As discussed above, nerve endings innervating the carotid sinus are comprised of both arterial baroreceptors and arterial chemoreceptors (Seagard et al., 1990; Porzionato et al., 2019). In fact, retrograde labeling of carotid sinus sensory nerve endings revealed that only 15% of the labeled neurons expressed the mechanically gated ion-channels *Piezo1* and *Piezo2* (Zeng et al., 2018). Retrogradely labeled carotid sinus neurons that were *Piezo* negative were presumed to be chemoreceptors (Zeng et al., 2018). This indicates that most sensory nerve endings innervating the carotid sinus are likely to be arterial chemoreceptors. However, sensory nerve endings innervating the aortic arch are primarily arterial baroreceptors. The soma of sensory nerve endings innervating the aortic arch reside within the nodose ganglion of the vagus nerve (Min et al., 2019; Elsaafien et al., 2022; Scott et al., 2025). Delivering a cre-dependent adeno-associated viral vector (AAV) to the nodose ganglion of *Piezo2-ires-Cre* mice labels *Piezo2*-containing vagal afferents, which were observed to innervate the aortic arch (Min et al., 2019). Whereas delivering the viral vector to the nodose ganglion of *Gpr65-ires-Cre* mice reveals no nerve endings at the aortic arch (Min et al., 2019). Proton-sensing G-protein coupled receptor 65 (Gpr65) is a chemosensor thought to be implicated in arterial chemoreception (Yu et al., 2025). These studies indicate that nerve endings innervating the aortic arch are mainly arterial baroreceptors. Thus, to selectively investigate arterial baroreceptors an “organ-specific” approach that targets arterial baroreceptors at the aortic arch, termed as aortic baroreceptors, is needed. In the current review, we will focus on discussing aortic baroreceptors.

### 3.1 Anatomy and physiology of aortic baroreceptors

We have recently developed a selective and organ-specific approach to target and label aortic baroreceptors (Elsaafien et al., 2022). A retrograde AAV directing the expression of the fluorophore tdTomato is directly applied to the aortic arch at the site of the baroreceptor-containing nerve endings (Elsaafien et al., 2022). Baroreceptor-sensitive nerve endings have distinct morphology described as end-net terminals and flower-spray endings (Cheng et al., 1997; Min et al., 2019). They innervate the vascular wall of the aortic arch, and they are located inferior to where the left common carotid artery and the left subclavian artery bifurcate form the aortic arch and caudally near the arterial ligament (Min et al., 2019; Elsaafien et al., 2022). These endings innervate both the ventral and dorsal surface of the aortic arch giving rise to neuronal fibers that join the aortic depressor nerve, forming a saddle-like structure or an “aortic claw” that monitors stretch at the aortic arch (Min et al., 2019; Elsaafien et al., 2022). These fibers travel through the aortic depressor nerve and join the vagus nerve through the superior laryngeal nerve. The soma resides within the nodose ganglion of the vagus nerve. The nodose ganglion of the vagus nerve houses several pseudounipolar neurons, which are the primary sensory neurons of the peripheral nervous system. These neurons are characterized by having a cell body residing within the nodose ganglion. The soma gives rise to two distinct axons, a peripheral axon which innervates peripheral organs and a central axon that terminates in the hindbrain (Berthoud and Neuhuber, 2000; Han et al., 2018; Bai et al., 2019; Kupari et al., 2019). This organization allows peripheral sensory vagal afferents to monitor internal bodily state and convey these signals to the hindbrain. In the case of aortic baroreceptors, stretch is monitored and is conveyed to the nucleus of the solitary tract (NTS) of the brainstem. Specifically, aortic baroreceptors terminate in the caudal and intermediate portions of the NTS (Cottle, 1964; Lipski et al., 1975; Mendelowitz et al., 1992; Elsaafien et al., 2022; Scott et al., 2025). Whereas arterial chemoreceptors have nerve endings characterized as pericellular endings innervating small cell bodies (Cheng et al., 1997), found abundantly in the carotid sinus and terminate in the most caudal portion of the NTS (Colombari and Talman, 1995; Colombari and Menani, 1996). Targeting arterial baroreceptors at the aortic arch revealed that labeled nerve endings have the morphology of baroreceptor-sensitive nerve endings and not chemoreceptor-sensitive endings (Elsaafien et al., 2022). Furthermore, the optogenetic stimulation of aortic baroreceptors mediated reductions in BP and HR (Elsaafien et al., 2022). Increased BP stretches the vascular wall of the aortic arch, which activates stretch sensitive aortic baroreceptors that innervate the aortic arch (Baumer-Harrison et al., 2024). Once activated, stretch is converted into action potentials conveyed through aortic baroreceptor afferent axons into the NTS. These axons release glutamate to activate second order neurons with the NTS (Elsaafien et al., 2022). Subsequently, SNA is inhibited, reducing total peripheral resistance, promoting vasodilation and reducing cardiac output by decreasing HR and stroke volume (Ismay et al., 1979; Lee et al., 1980; Guo and Abboud, 1984). Additionally, parasympathetic nerve activity is increased leading to reduced cardiac output. Ultimately, both total peripheral resistance and cardiac output are reduced through reduced SNA and increased parasympathetic nerve activity (Ismay et al., 1979; Lee et al., 1980; Guo and Abboud, 1984). Thus, increased BP activates stretch sensitive aortic baroreceptors which mediate reductions in BP and cardiac output that ultimately restore BP to homeostatic levels. The physiological responses mediated by the baroreflex are commonly assessed by pharmacological challenges (Ismay et al., 1979; Lee et al., 1980; Guo and Abboud, 1984; Bishop and Sanderford, 2000; Sanderford and Bishop, 2000, 2002). For a pressor challenge that overloads the baroreflex, the α-adrenergic receptor agonist phenylephrine is administered intravenously to induce vasoconstriction that subsequently increases BP. Increased BP by phenylephrine activates/overloads the baroreflex (observed as an increase in aortic depressor nerve activity), leading to sympatho-inhibition (observed as a decrease in lumbar or renal SNA), evoking a reduction in HR (Stocker et al., 2019). In contrast, sodium nitroprusside, a potent vasodilator serves as a depressor challenge that unloads the baroreflex, and has the opposite effect to phenylephrine (Stocker et al., 2019). Comparing changes in BP, HR, and SNA during pressor and depressor challenges is used to assess baroreflex sensitivity (Bishop and Sanderford, 2000; Sanderford and Bishop, 2000, 2002). For example, studies used baroreflex challenges to demonstrate reduced baroreflex sensitivity in rodent models of hypertension (Sanderford and Bishop, 2000). Thus, stretch sensation at the aortic arch mediates the regulation of BP, and impairments may lead to the development of hypertension. Overall, targeting arterial baroreceptors at the aortic arch allows for selectively investigating stretch-sensitive aortic baroreceptors (Elsaafien et al., 2022). Utilizing an organ-specific approach can lead to unraveling the molecular, structural, and functional phenotype of arterial baroreceptors in healthy and disease state. In fact, follow up studies demonstrate that aortic baroreceptors not only utilize *Piezo* channels, but they also engage ENaC and TRPA1 channels to evoke a baroreflex response (Elsaafien et al., 2025). This raises the question of the identity of stretch sensors utilized by aortic baroreceptors and the role they play in the development of hypertension.

### 3.2 Stretch sensors in blood pressure regulation

A stretch sensor is an ion channel that opens following mechanical forces that stretch the cellular membrane (Gomis et al., 2008; Dong et al., 2010; Ranade et al., 2014; Woo et al., 2014). This allows for the influx of calcium ions which depolarizes the cell (Gomis et al., 2008; Dong et al., 2010; Ranade et al., 2014; Woo et al., 2014). In the case of aortic baroreceptors, stretch sensors convert stretch into electrical signals or action potentials conveyed to the brainstem (Sun et al., 2009; Lau O. C. et al., 2016; Zeng et al., 2018; Yan et al., 2021). Several ion channels have been implicated in sensing vascular stretch (Drummond et al., 1998). Mechanically gated ion channels *Piezo1* and *Piezo2* are expressed on aortic baroreceptors (Min et al., 2019; Elsaafien et al., 2022; Scott et al., 2023), involved in vascular stretch sensation (Chesler et al., 2016; Murthy et al., 2017) and have been implicated in evoking the baroreflex response (Zeng et al., 2018; Min et al., 2019; Elsaafien et al., 2022). In addition, several members of transient receptor potential channel family have been implicated in baroreception. For example recent studies have demonstrated that aortic baroreceptors express transient receptor potential ankyrin 1 (TRPA1) channels to engage the baroreflex (Scott et al., 2023; Elsaafien et al., 2025). Furthermore, previous studies have demonstrated the involvement of transient receptor potential vanilloid 1 (TRPV1) (Sun et al., 2009; Yan et al., 2021), transient receptor potential canonical 5 (TRPC5) (Lau et al., 2018a,b; Thakore et al., 2018), and sodium epithelial channels (ENaC) (Elsaafien et al., 2025) in sensing stretch at aortic baroreceptors to evoke the baroreflex. In the next section, several ion channels and their involvement in sensing vascular stretch in aortic baroreceptors to evoke a baroreflex response will be discussed.

#### 3.2.1 Mechanically gated ion channels: Piezo1 and Piezo2

*Piezo* channels are mechanically gated ion channels discovered and described in 2010 by Nobel Prize Laureate Ardem Patapoutian (Coste et al., 2010, 2012, 2015; Ge et al., 2015). There are two different types of *Piezo* channels; *Piezo1* and *Piezo2* (Coste et al., 2010). They are non-selective cation channels with low permeability to chloride (Coste et al., 2010, 2012, 2015). The ion channel opens following stretch at the cellular membrane, allowing for calcium to flow into the cell (Ranade et al., 2014; Woo et al., 2014; Murthy et al., 2017). *Piezo* channels have been implicated in cardiovascular function and disease (Coste and Delmas, 2024). They were first described as being necessary for stretch sensing in endothelial cells to regulate vascular tone and BP in response to fluid shear stress (Wang et al., 2016). Subsequently, *Piezo1* and *Piezo2* transcripts were found to be expressed in the nodose ganglion of the vagus nerve (Zeng et al., 2018). These transcripts were found to colocalize to nodose ganglion neurons that innervate the aortic arch (Elsaafien et al., 2022). Indeed, delivering a Cre-dependent AAV to the nodose ganglion of *Piezo2-ires-Cre* mice revealed that aortic baroreceptors utilize *Piezo* channels in baroreception (Min et al., 2019). The selective optogenetic stimulation of aortic baroreceptors that contain *Piezo* channels evokes a baroreflex response that is observed as a reduction in BP and HR (Zeng et al., 2018; Min et al., 2019; Elsaafien et al., 2022). This suggests that *Piezo* channels are engaged by aortic baroreceptors to evoke the baroreflex. In addition, the selective ablation of aortic baroreceptors that express *Piezo2* channels impaired baroreflex sensitivity in response to a baroreflex challenge (Min et al., 2019). Furthermore, the double knockout of *Piezo1* and *Piezo2* channels resulted in impairment of the baroreflex (Zeng et al., 2018). These studies suggest that *Piezo* channels expressed by aortic baroreceptors are both sufficient and necessary for regulating changes in BP and engaging the baroreflex. This raises the question of whether *Piezo* channels are the exclusive stretch sensors utilized by aortic baroreceptors. Unequivocally, not all aortic baroreceptors express *Piezo* channels (Stocker et al., 2019; Elsaafien et al., 2022). In fact there is a plethora of studies suggesting a role for various ion channels in stretch sensation and baroreception (Lu et al., 2009; Sun et al., 2009; Lau et al., 2018a,b; Thakore et al., 2018; Yan et al., 2021; Scott et al., 2023). Thus, one can conclude that aortic baroreceptors utilize a variety of ion channels in sensing vascular stretch to evoke the baroreflex and that no single channel is exclusively responsible for stretch sensation by aortic baroreceptors (Yang et al., 2022). This may raise the concern that stretch sensors engaged by aortic baroreceptors are redundant. Conceivably, specific stretch sensors may be involved in priming the function of other stretch sensors during baroreception, some may interact with specific receptors and endocrine factors, some may be involved in dynamic transmission by being expressed on myelinated A-fiber vagal afferents involved in transient BP changes (Seagard et al., 1993), whereas others may be implicated in tonic transmission through unmyelinated C-fibers that are involved in sustained BP changes (Fan et al., 1996; Fan and Andresen, 1998; Armstrong and Moore, 2023). Overall, baroreception is a complex physiological reflex that involves different fibers and stretch sensors to evoke, prime, maintain and fine tune the reflex to achieve BP homeostasis under many different physiological conditions.

#### 3.2.2 Transient receptor potential channels: TRPV1, TRPC5, TRPA1 and TRPV4

The transient receptor potential (TRP) superfamily consists of 28 channels divided into six subfamilies; vanilloid (TRPV), canonical (TRPC), ankyrin (TRPA), melastatin (TRPM), mucolipin (TRPML), and polycystin (TRPP) (Samanta et al., 2018). TRP channels are cation channels that play a role in mediating membrane potential (Dong et al., 2010). Different subfamilies are activated by different mechanical and chemical stimuli. For example, TRPC5 channels are activated by stretch (Gomis et al., 2008), whereas TRPV1 channels are activated by nociceptive molecules such as heat (Caterina et al., 1997; Tominaga et al., 1998), ischemia (Zahner et al., 2003; Pan and Chen, 2004) and changes in fluid osmolarity (Ciura and Bourque, 2006; Naeini et al., 2006). Activation of these channels leads calcium influx and subsequently regulating membrane potential (Gomis et al., 2008; Dong et al., 2010). Interestingly, a role for TRP channels in stretch sensation was first described in 1997, long before *Piezo* channels were discovered (Colbert and Smith, 1997). TRP-like channels were found to be encoded by the osm-9 gene that is involved in detecting mechanical stimuli in nematodes (Colbert and Smith, 1997). In mammals, the mechanosensory function of osm-9 gene is replaced with the functional ortholog TRPV4 gene (Liedtke et al., 2003). In addition, TRP channels, such as TRPC5 and TRPV1, were found to be expressed by aortic baroreceptors to engage the baroreflex as early as 2005 (Glazebrook et al., 2005; Sun et al., 2009). Thus far several TRP channels have been described as ion channels activated by mechanical stretch, inducing TRPA1, TRPV1/V2, TRPC1/C3/C5/C6, TRPM4/M7 and TRPPP1/PP2 channels (Yang et al., 2022). In the following section, the role TRPV1, TRPC5, TRPA1 and TRPV4 channels play in sensing vascular stretch in baroreception will be discussed.

##### 3.2.2.1 Transient receptor potential vanilloid 1 (TRPV1)

TRPV1 channels have been extensively studied in pain modulation and nociception (Palazzo et al., 2008; Riera et al., 2014; Mobasheri et al., 2024). In fact, TRPV1 channels are expressed by sensory neurons of the dorsal root ganglion (Gallego-Sandín et al., 2009; Berta et al., 2017; Zhang et al., 2021; Chen et al., 2022). These neurons are activated by nociceptive stimuli and are involved in conveying pain signals to the brain (Caterina et al., 1997; Tominaga et al., 1998; Zahner et al., 2003; Pan and Chen, 2004; Ciura and Bourque, 2006; Naeini et al., 2006). Interestingly, TRPV1 expressing sensory afferents that innervate the kidney, termed as afferent renal nerves, have been implicated in stretch sensing in response to increased intrarenal pelvic pressure (Walder et al., 2012). Afferent renal neurons express TRPV1 channels (Stocker et al., 2025). In fact within T11-L1 dorsal root ganglion, ~60% of renal sensory neurons retrogradely labeled from the kidney express TRPV1 channels (Stocker and Sullivan, 2023; Stocker et al., 2025). Interestingly, these neurons are sensitive to changes in renal artery pressure and not to pelvic pressure (Sullivan et al., 2025). Increased pressure within the renal artery, due to ischemia or occlusion, increases the activity of TRPV1-expressing afferent renal nerve activity, subsequently increasing renal SNA and BP (Ong et al., 2019; Sullivan et al., 2025). Fascinatingly, the global knockout of TRPV1 channels attenuates sympatho-excitation and increased BP in a renovascular model of hypertension (Stocker and Sullivan, 2023). However, the selective knockout of TRPV1 channels from sensory renal afferents does not attenuate hypertension in the same animal model (Stocker et al., 2025). These studies indicate that TRPV1 channels are involved in sensing vascular stretch, regulating SNA and BP, and the global knockout of TRPV1 channels attenuates the hypertensive phenotype. However, their implication in the hypertensive phenotype is independent of renal sensory afferents. Thus, raising the question of whether TRPV1 channels are involved in baroreception and the development of hypertension by signaling through aortic baroreceptors. Previous studies indicate that baroreflex sensitivity is impaired in the presence of TRPV1 blockers (Sun et al., 2009; Yu et al., 2017) and that TRPV1 knockout blunts the baroreflex (Zhong et al., 2019). Furthermore, TRPV1 channels mediate reflex cardiovascular responses through both myelinated A-fiber and unmyelinated C-fibers (Longhurst et al., 1984). Intriguingly, TRPV1 channels are expressed on nerve ending innervating the aortic arch, neuronal soma residing within the nodose ganglion of the vagus and afferent fibers terminating in the NTS (Sun et al., 2009). Thus, TRPV1 channels are expressed by aortic baroreceptors. The selective ablation of TRPV1 expressing aortic baroreceptors impairs baroreflex function as demonstrated by the loss of sympatho-inhibition and bradycardia in response to a pressor challenge (Sun et al., 2009). Thus, TRPV1 channels are a primary candidate for a stretch sensor engaged by aortic baroreceptors. However, the precise role they play in baroreception remains to be fully characterized.

##### 3.2.2.2 Transient receptor potential canonical 5 (TRPC5)

TRPC5 channels have been extensively studied for the role they play in regulating cardiovascular physiology. These channels are activated by hypo-osmolality and membrane stretch (Gomis et al., 2008). They are widely expressed on cardiovascular tissue such as cardiac ventricles, endothelial cells and smooth muscle cells (Jiang et al., 2011). Interestingly, their activity has been linked to atherosclerosis, heart failure and elevated BP (Bush et al., 2006; Xiao et al., 2017; Wen et al., 2020). The mRNA and protein levels of TRPC5 channels in the heart are elevated during heart failure (Bush et al., 2006). In heart failure, irregular mechanical forces activate cardiac TRPC5 channels, subsequently leading to calcium-dependent maladaptive heart remodeling (Bush et al., 2006). Thus, a role for TRPC5 channels in stretch dependent cardiovascular regulation is well characterized. In fact, TRPC5 channels were found to be expressed by aortic baroreceptors long before *Piezo* channels were discovered. TRPC5 channels are expressed within the nerve endings, soma, A- and C-fibers of nodose ganglion neurons that innervate the aortic arch (Glazebrook et al., 2005). Patch clamp electrophysiology of aortic baroreceptors isolated from the nodose ganglion of rats, revealed that stretch increased neuronal activity in aortic baroreceptor (Lau O. C. et al., 2016). Inhibiting TRPC5 in aortic baroreceptors completely abolished the neuronal activity evoked by stretch (Lau O. C. et al., 2016). Furthermore, global knockout of TRPC5 channels impaired baroreflex sensitivity and increased BP in conscious freely moving mice (Lau O. C. et al., 2016; Lau et al., 2018a,b). Finally, the ablation of TRPC5 from sensory nodose ganglion neurons abolished stretch sensitivity in aortic baroreceptors, discharge of action potentials in the aortic depressor nerve and reduced baroreflex sensitivity (Lau O. C. et al., 2016). Altogether, these studies support a role for TRPC5 channels in stretch sensation by aortic baroreceptors that mediate baroreception.

##### 3.2.2.3 Transient receptor potential ankyrin 1 (TRPA1)

TRPA1 channels have been linked to regulating BP and the cardiovascular system (Wang et al., 2019). For example, intravenous administration of the TRPA1 agonist, acrolein, induces vasodilation and reduces BP and HR (Pozsgai et al., 2010; Earley, 2012). In addition, activating TRPA1 channels mediates changes in cardiac mechanical function, and heart rate variability characterized by autonomic imbalance (Kurhanewicz et al., 2017, 2018). This raises the question of whether TRPA1 channels are expressed by autonomic neurons. Indeed, TRPA1 channels were found to be expressed on cardiac sympathetic afferents and their stimulation leads to modulating renal SNA, BP and HR (Adam et al., 2019). Intriguingly, the expression of TRPA1 channels on cardiac sympathetic afferents is reduced in chronic heart failure (Adam et al., 2019). Furthermore, the levels of TRPA1 channels in the kidneys are decreased in angiotensin-II mediated hypertension, and the knockout of TRPA1 channels exacerbates angiotensin II-induced kidney injury (Ma et al., 2019). These studies not only suggest a role of TRPA1 in regulating cardiovascular physiology, but they indicate a role for TRPA1 channels in afferent signaling that modulates autonomic outflow. This makes TRPA1 channels a candidate for stretch sensation in aortic baroreceptors that evoke baroreflex responses. Indeed, TRPA1 channels were found to be expressed by aortic baroreceptors (Scott et al., 2025) and engaging them is involved in baroreception (Elsaafien et al., 2025). However, further research investigating the precise role TRPA1 channels play in baroreception is warranted.

##### 3.2.2.4 Transient receptor potential vanilloid 4 (TRPV4)

TRPV4 channels are activated by mechanical stimuli that includes, cell swelling (Li et al., 2003; Strotmann et al., 2000; Liedtke et al., 2003; Nilius, 2009), stretch (Gao et al., 2003; Hartmannsgruber et al., 2007), and viscous loading (Andrade et al., 2005). Recent studies suggest that TRPV4 channels are implicated in regulating cardiovascular physiology and disease (Randhawa and Jaggi, 2015). Vascular endothelium and smooth muscle cell express TRPV4 channels to regulate vasomotor tone (Earley et al., 2009; Chen and Li, 2021; Zhu et al., 2023). Activation of TRPV4 channels on smooth muscle cells leads to calcium influx that induces nitric oxide production in smooth muscle cells and subsequently evokes vasodilation (Chen and Li, 2021). Interestingly, vasodilation is blunted in TRPV4 knockout mice (Earley et al., 2009). In addition, hypertension induced by nitric oxide inhibition (Earley et al., 2009) or angiotensin-II (Nishijima et al., 2014) is exacerbated in TRPV4 knockout mice. Fascinatingly, a role for TRPV4 channels in sensing vascular stretch to modulate BP has been well characterized in the kidneys (Seghers et al., 2016). Juxtaglomerular cells of the kidneys sense perfusion pressure at the afferent renal arteriole and secrete renin in response to low perfusion pressure (MacGregor et al., 1981; Friis et al., 2013). Secretion of renin in systemic circulation leads to the production of angiotensin-II which induces vasoconstriction to increase perfusion pressure and BP (MacGregor et al., 1981; Friis et al., 2013). TRPV4 channels are expressed by juxtaglomerular cells and are activated in response to increased BP, which increases stretch at the afferent renal arteriole (Seghers et al., 2016). Activation of TRPV4 channels leads to calcium influx into juxtaglomerular cells which subsequently inhibits renin secretion, and reducing systemic angiotensin-II levels to inhibit vasoconstriction (Seghers et al., 2016). This acts as a classical negative feedback mechanism to maintain BP at homeostatic levels through actions on the kidney and the renin-angiotensin system. Thus, raising the question of whether TRPV4 channels are utilized by aortic baroreceptors to evoke the baroreflex and maintain BP at homeostatic levels. Interestingly, a previous study has shown that TRPV4 channels are expressed within the nodose ganglion and may be involved in baroreflex responses (Lau E. O. C. et al., 2016). Although TRPV4 channels may be a candidate for a stretch sensor utilized by aortic baroreceptors, studies characterizing their expression on aortic baroreceptors and the role they play in baroreception are needed.

#### 3.2.3 Acid sensing ion channels (ASICs)

ASICs are channels gated by extracellular acidosis and changes in pH activates these channels (Collier and Snyder, 2009). ASICs are low pH-activated Na^+^-permeable ion channels that widely expressed in the peripheral and central nervous system (Vullo and Kellenberger, 2020). There are four different types of ASIC channels: ASIC1, ASIC2, ASIC3, and ASIC 4 (Vullo and Kellenberger, 2020). Interestingly, transcripts for ASIC1, 2, and 3 are expressed within the nodose ganglion of the vagus nerve (Lu et al., 2009). ASIC2 appears to be the least acid sensitive (Benson et al., 2002) and is involved in stretch sensation (Price et al., 2000; Page et al., 2005). For example, ASIC2 channels are required for stretch sensation in nodose ganglion neurons that innervate the gastrointestinal tract (Page et al., 2005). Furthermore, stretch sensation by ASIC2 channels is implicated in regulating cardiovascular function and disease (Gannon et al., 2008; Lu et al., 2022). ASIC2 knockout impairs stretch-induced vasoconstriction in vascular smooth muscle cells in the renal afferent arteriole (Gannon et al., 2008) and in middle cerebral arteries (Lu et al., 2022). Thus, raising the question of whether ASIC2 channels are implicated in stretch sensation in aortic baroreceptors. Indeed, ASIC2 channels are expressed within sensory neurons of the nodose ganglion and nerve endings that innervate the aortic arch (Lu et al., 2009). These channels are required by aortic baroreceptors for the sensation of vascular stretch that is converted into action potentials and conveyed by the aortic depressor nerve to evoke the baroreflex response (Lu et al., 2009). The knockout of ASIC2 channels impairs baroreflex sensitivity which leads to elevated sympathetic and suppressed parasympathetic nerve activity, subsequently leading to an increased BP and HR (Lu et al., 2009). Thus, ASIC2 channels are required for stretch sensation by aortic baroreceptors to evoke the baroreflex, and the knockout of ASICS2 channels leads to the development of a hypertensive phenotype (Lu et al., 2009). Interestingly, ASIC2 channels are involved in mediating stretch sensation by interacting with other stretch sensors such as TRPV1 channels (Yan et al., 2021) and epithelial sodium channels (Lu et al., 2022). The role of ASIC2 in stretch-mediated baroreflex responses and by interacting with other stretch sensors warrants further investigation.

#### 3.2.4 Epithelial sodium channels (ENaCs)

ENaCs were discovered in 1993 as amiloride sensitive channels in the colon of salt-deprived rats (Canessa et al., 1993). In vertebrates, they form heterodimers consisting of α, β, γ subunits and are known to be activated through stretch at the cellular membrane (Baldin et al., 2020). These channels are implicated in fluid and sodium transport across organs such as the lungs, pancreas, kidney, liver, and colon (McDonald et al., 1994). Interestingly, ENaC activity is modulated by a number of hormones that regulate fluid balance and BP, such as angiotensin-II, aldosterone, and vasopressin (Crofton et al., 1978; Share and Crofton, 1982; Tomita et al., 1985; Schafer and Hawk, 1992; Bankir et al., 2010; Li and Fung, 2019). This suggests that ENaCs may be implicated in regulating cardiovascular function and disease. For example, ENaCs are expressed by vascular smooth muscle cells are required for pressure-induced vasoconstriction of the renal afferent arteriole (Lu et al., 2022). Furthermore, previous studies have demonstrated that the β and γ subunit expression within the aortic arch and nodose ganglion is reduced on in chronic heart failure in mice (Li et al., 2016). This raises the question of whether ENaC channels are implicated in stretch sensation by aortic baroreceptors to regulate BP. ENaC channels are found to be expressed within nerve ending and soma of nodose ganglion neurons that innervate the aortic arch (Drummond et al., 1998), suggesting that aortic baroreceptors may utilize ENaC channels to elicit reflex regulation of BP. Intriguingly, mechanical stretch increased calcium influx within aortic baroreceptors which was abolished following the pretreatment with the ENaC channel inhibitor, amiloride (Drummond et al., 1998). The inhibition of ENaC channels impaired reflex regulation of BP (Drummond et al., 1998). These studies suggest that ENaC channels are engaged by aortic baroreceptors in sensing vascular stretch to evoke reflex regulation of BP (Drummond et al., 1998; Elsaafien et al., 2025). The precise role of ENaC channels in baroreception and the development of hypertension remains to be interrogated.

#### 3.2.5 Aquaporin channels (AQP)

AQP channels are membrane proteins implicated in transporting water across the cellular membrane of epithelia and endothelia in response to osmotic gradients in several organs including the kidneys, eyes, lungs, and gastrointestinal system (Verkman, 2013). There are currently 46 types of AQP across several subfamilies (Heymann and Engel, 1999). Of these AQP channels, humans express AQP0, AQP1, AQP2, AQP3, AQP4, AQP5, and AQP6 with more being found in other animals, plants, and bacteria (Heymann and Engel, 1999). As these channels are involved in water transport and therefore cell volume, they are thought to be implicated in sensing stretch at the cellular membrane. Regulatory volume decreasing (RVD) is a mechanism implicated in the maintenance of cell volume to counteract cellular swelling in hypotonic conditions by reducing cell volume (Okada et al., 2001). Several previous studies have distinguished AQP channels as key players in RVD (Kuang et al., 2004; Ford et al., 2005; Kida et al., 2005; Liu et al., 2006; Benfenati et al., 2011; Chen and Duan, 2011; Galizia et al., 2012). Thus, understanding RVD is essential in investigating whether AQP channels are implicated in stretch sensation. In theory, for the cell to initiate RVD due to swelling it would have to detect when and by how much it is being stretched. Interestingly, AQP channels interact with other ion channels that are implicated in stretch sensation, to evoke an RVD response (Verkman, 2013). For example, AQP4 forms a complex with TRPV4, a candidate for vascular stretch sensing by aortic baroreceptors (Lau E. O. C. et al., 2016), where TRPV4 channels are required to activate AQP4 (Benfenati et al., 2011). Interestingly, astrocytes expressing both AQP4 and TRPV4 channels respond to hypotonic stress by increased calcium influx which initiates an RVD response (Benfenati et al., 2011). The knockout of TRPV4 and AQP4 in astrocytes impairs the initiation of RVD in response to a hypotonic challenge (Benfenati et al., 2011). Intriguingly, functional studies reveal that calcium influx is mediated through TRPV4 and not AQP4 channels, however the knockout of AQP4 impairs the RVD responses (Benfenati et al., 2011). Fascinatingly, this interaction is specific to AQP4 and no other AQP channels. Thus, suggesting an interaction between AQP4 and TRPV4 channels to detect and evoke a response to a hypotonic challenge. Further studies support this interaction, as TRPV4 interaction with AQP5 in salivary glands (Liu et al., 2006) and AQP2 in renal cells (Galizia et al., 2012) are necessary for the cell to mediate an RVD in response to cellular swelling. Given that TRPV4 and member of the TRP super family are expressed by aortic baroreceptors and are involved in stretch sensing (Sun et al., 2009; Lau E. O. C. et al., 2016; Lau O. C. et al., 2016; Zhong et al., 2019; Elsaafien et al., 2025; Scott et al., 2025), and that AQP channels sense cellular swelling to engage the RVD response (Okada et al., 2001; Benfenati et al., 2011), we speculate that AQP channels may be involved in stretch sensing utilized by aortic baroreceptors. To this end, several AQP channels have been found in the peripheral nervous system, such as in neurons of the trigeminal ganglion, dorsal root ganglion, and the nodose ganglion of the vagus nerve (Ma et al., 2011). Thus, AQP channels are possible candidates for stretch sensing and interacting with other stretch sensors in aortic baroreceptors. However, whether they are expressed by aortic baroreceptors to detect stretch and interact with other stretch sensors to evoke reflex control of BP, remains to be elucidated.

## 4 Baroreflex insensitivity in hypertension: role for estradiol?

Although the involvement of the baroreflex in hypertension is debatable, there is a plethora of literature linking baroreflex impairment to the development of hypertension. In 2005 a case report was published in the American Heart Association's Hypertension Grand Rounds (Heusser et al., 2005). The report presents two patients with “baroreflex failure” diagnosed following neck injuries. Both patients exhibited volatile hypertension with systolic readings up to 300 mmHg, where hypertension is further exacerbated by a sympathetic stimuli such as, stress, danger or exercise (Heusser et al., 2005). This case report unequivocally links baroreflex impairment to the development of hypertension. In addition, *in vivo* animal studies utilizing two photon intravital imaging of arterial baroreceptors revealed that hypertension impairs baroreceptor's abilities to respond to changes in BP (Baumer-Harrison et al., 2024). Furthermore, baroreflex insensitivity or impairment has been reported as the hallmark of many animal models of hypertension (Nakamura et al., 1988; Ueno et al., 1988; Grassi et al., 2014; Mancia and Grassi, 2014). The implication of baroreflex insensitivity in hypertension will be discussed in the following section.

### 4.1 Baroreflex impairment precedes the onset of hypertension

Reduced baroreflex sensitivity impairs reflex regulation of SNA and BP. Previous studies found that baroreflex insensitivity leads to impairment in sympatho-inhibition in response to increased BP (Ismay et al., 1979; Lee et al., 1980; Guo and Abboud, 1984). For example, many studies utilizing nerve recording from renal and lumbar SNA during a pressor baroreflex challenge have linked baroreflex insensitivity to over-activity of SNA in rodent models of hypertension (Bishop and Sanderford, 2000; Sanderford and Bishop, 2000, 2002). Angiotensin-II mediated hypertension attenuates reflex inhibition of lumbar SNA and bradycardia in response to the infusion of phenylephrine (Ismay et al., 1979; Lee et al., 1980; Guo and Abboud, 1984). Findings from these studies indicate that baroreflex impairment diminishes the abilities of baroreceptors to inhibit sympathetic outflow, subsequently leading to elevated sympathetic nerve activity and hypertension. Indeed, maximum SNA in response to phenylephrine administration is increased by 50% in angiotensin-II mediated hypertension compared to normotensive control groups (Bishop and Sanderford, 2000; Sanderford and Bishop, 2000, 2002). These findings in animal models echo the case report mentioned above, in that baroreflex failure leads to impairments in reflex inhibition of sympathetic outflow leading to elevated SNA and subsequently sympathetic stimuli exacerbates and contributes to the hypertensive phenotype. Intriguingly, studies demonstrate that baroreflex impairment precedes sustained elevations in SNA which in turn precedes chronic elevations in BP and the development of hypertension (Oliveira-Sales et al., 2014, 2016). These studies were conducted in the Goldblatt or the 2-kidney 1-clip (2K1C) renovascular model of hypertension in rats to reveal the implication of the autonomic nervous system in the development and maintenance of hypertension (Katholi et al., 1982; Oliveira-Sales et al., 2006; Peotta et al., 2007; Souza et al., 2008; Zhu et al., 2009; Oliveira-Sales et al., 2014, 2016). The Goldblatt model is a unique model of hypertension as it offers the temporal resolution to study and investigate the development of hypertension (Goldblatt et al., 1934; Navar et al., 1998). Blood flow to the left kidney is chronically reduced through occlusion by placing a clip on the left renal artery to constrict blood flow, whereas blood flow to the right kidney remains intact, hence the name 2-kidney 1-clip (2K1C) (Goldblatt et al., 1934; Navar et al., 1998). As blood flow is reduced to the left kidney, renin is secreted into circulation which leads to the production of angiotensin-II, as an effort for the kidney to restore blood flow (Goldblatt et al., 1934; Navar et al., 1998). However, as a clip is permanently placed on the left renal artery, blood flow is not restored, leading to a systemic spill-over of renin-angiotensin. This overloads systemic circulation with angiotensin-II and subsequently leads to the development of hypertension, usually occurring around the 4^th^-5^th^ week following renal artery clipping (Goldblatt et al., 1934; Navar et al., 1998; Oliveira-Sales et al., 2014). Thus, allowing for investigating the mechanisms that contribute to the development of hypertension. Baroreflex sensitivity (BRS) is reduced immediately following left renal artery clipping (Oliveira-Sales et al., 2014). Recording thoracic SNA revealed that elevations in sympathetic outflow occurs three weeks following left renal artery clipping (Oliveira-Sales et al., 2016). The onset of BP elevations occurs around the 3^rd^ week after clipping and plateaus at 185 mmHg five weeks following left renal artery clipping (Oliveira-Sales et al., 2014). These studies indicate that baroreflex insensitivity precedes sympathetic overactivity which occurs prior to sustained elevations in BP and the development of hypertension. As the prevalence of hypertension differs amongst men and women (Fryar et al., 2024), we raise the question of whether baroreflex insensitivity contributes to sex-specific differences in hypertension?

### 4.2 Interplay between baroreception, estradiol and hypertension

Cardiovascular disease affects men and women differently (Fryar et al., 2024). The incidence and severity of hypertension is lower amongst women than in men (Kotchen et al., 1982; Schenck-Gustafsson, 1996; Dubey et al., 2002). Epidemiological data obtained between August 2021–August 2023 suggest that the incidence of hypertension in young adults (ages 18–39 years old) to be ~30% in men and ~16% in women (Fryar et al., 2024). Intriguingly, the incidence increases in older adults (ages 60 years and older) to ~72% in men and ~70% in women. Investigating sex differences is crucial in understanding mechanisms driving the pathogenesis of hypertension. In clinical studies, reduced baroreflex sensitivity (BRS) has been associated with hypertension (Bristow et al., 1969; Kardos et al., 2001; Indumathy et al., 2015). Assessing BRS in human subjects revealed that BRS is reduced by at least 6-fold in hypertensive patients compared to healthy normotensive subjects without hypertension (Bristow et al., 1969). In fact, both age and sex contribute to BRS, in that BRS is reduced with age (Man et al., 2021). Interestingly, women exhibit enhanced BRS compared to men, suggesting that women are more sensitive to changes and regulating BP than men (Tanaka et al., 2003). Intriguingly, BRS changes during the menstrual cycle in women, where BRS is enhanced during the pre-ovulation phase (Tanaka et al., 2003). This suggests that estradiol may play a role in enhancing BRS and subsequently improving BP control and regulation. Indeed, a positive correlation between plasma estradiol levels and BRS was found across the menstrual cycle in women (Tanaka et al., 2003). These studies link circulating estradiol levels with enhanced BRS in women, and as estradiol levels are reduced during post-menopause BRS sensitivity is reduced, possibly explaining the difference in the incidence of hypertension between women and men. Further mechanistical studies in rodent models validates this association. Intriguingly, compared to age-matched male rats, female rats exhibit intrinsically higher BRS that improves baroreflex regulation of BP (Jing-ran et al., 2025). The chronic intravenous administration of 17β-estradiol in ovariectomized female rats enhanced BRS in response to both a pressor and a depressor challenge (Saleh et al., 2000c). Furthermore, 17β-estradiol reduced sympathetic tone and enhanced parasympathetic tone (Saleh et al., 2000a,b). Follow up studies with electrophysiological recordings from vagal parasympathetic nerve and renal sympathetic nerve, revealed that 17β-estradiol increases vagal parasympathetic nerve activity and reduces sympathetic nerve activity in female rats leading to an enhanced BRS (Saleh et al., 2000b). Finally, in conscious freely-behaving rats, ovariectomy reduced BRS in female rats, and 17β-estradiol replacement in ovariectomized rats enhanced BRS to similar levels observed in intact female rats (El-Mas and Abdel-Rahman, 1998). These studies provide insights into mechanisms by which estradiol enhances BRS by blunting sympathetic outflow and increasing parasympathetic nerve activity which improves reflex regulation of BP (El-Mas and Abdel-Rahman, 1998; He et al., 1998; Mohamed et al., 1999; Saleh and Connell, 1999; Saleh et al., 2000a,b,c). In that, in all these studies estradiol injection blunted the phenylephrine evoked pressor response. Thus, indicating that estradiol enhances BRS, leading to improved baroreflex regulation of BP which induces BP lowering effects in female rats (Saleh and Connell, 1999; Saleh et al., 2000a,b,c). In fact, in both genetic and induced animal models of hypertension, females exhibit lower BP compared to male animals, where hypertension develops more rapidly and severely in male animals than in female animals (Xue et al., 2005). This includes, dahl-salt sensitive rats (Rowland and Fregly, 1992; Crofton and Ota, 1993), deoxycorticosterone acetate-saline hypertension (Ouchi et al., 1987; Crofton and Share, 1997), spontaneously hypertensive rats (Masubuchi et al., 1982; Chen and Meng, 1991; Reckelhoff et al., 1998; Reckelhoff, 2001), two-kidney one-clip renovascular hypertension in rats (Okuniewski et al., 1998), and angiotensin-II induced hypertension in mice (Xue et al., 2005). Intriguingly, ovariectomy in female rats and mice attenuates the sex-difference observed in the different animal models of hypertension (Okuniewski et al., 1998; Xue et al., 2005). For example, chronic infusion of angiotensin-II increased BP by 35 mmHg in male mice compared to an increase of 7 mmHg in female mice (Xue et al., 2005). However ovariectomy followed by chronic angiotensin-II infusion increased BP by 23 mmHg in ovariectomized female mice (Xue et al., 2005). Conversely, ovariectomy in normotensive female control rats increases BP and estradiol administration attenuates this increase (Hernández et al., 2000). Although sexual-dimorphism observed in rodent models of hypertension and in humans can be attributed to many factors including salt sensitivity and renal sodium handling (Oloyo et al., 2019; Soliman et al., 2022; Stadt and Layton, 2025), the discussed studies indicate that circulating estradiol and BRS may contribute to the sex-differences observed in rodent models of hypertension. It is evident that ovariectomy in female mice attenuates sex-specific differences in the development of experimental models of hypertension (Okuniewski et al., 1998; Xue et al., 2005). Therefore, these studies indicate a protective role for estradiol against impaired BRS, and further indicate that estradiol supplementation can be cardioprotective (El-Mas and Abdel-Rahman, 1998; He et al., 1998; Mohamed et al., 1999; Saleh and Connell, 1999; Saleh et al., 2000a,b,c. In fact, clinical studies demonstrate that the reduced BRS and increased BP in post-menopausal women are alleviated following estradiol replacement therapy (Huikuri et al., 1996; Hunt et al., 2001). This raises the question of whether estrogen receptors are expressed by aortic baroreceptors allowing for circulating estradiol to modulate BRS, autonomic outflow, BP and ultimately hypertension?

### 4.3 Estrogen receptors expression in autonomic neurons

**Figure 1:**
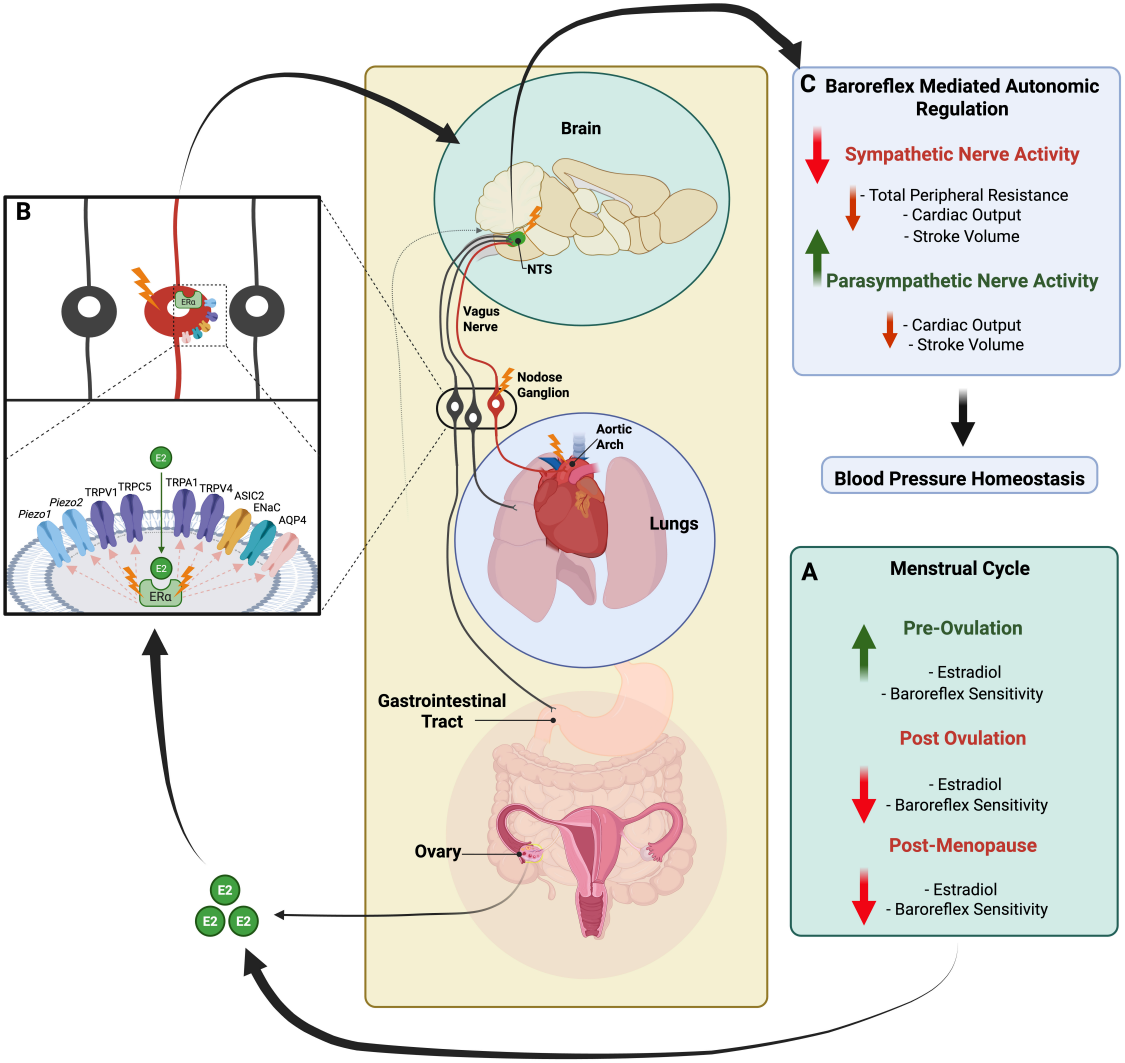
Working model: Estradiol engages stretch sensors in aortic baroreceptors to regulate the baroreflex. A schematic diagram depicting aortic baroreceptors innervating the aortic arch and terminating within the nucleus of the solitary tract (NTS). We propose that aortic baroreceptors may express estrogen receptor α and may engage several ion channels to detect vascular stretch. **(A)** During pre-ovulation the systemic levels of estradiol peak and drop post-ovulation and post-menopause. **(B)** Circulating estradiol modulates the activity of stretch sensors expressed by aortic baroreceptors through actions on estrogen receptor α (ERα). **(C)** This enhancement of baroreflex sensitivity suppresses sympathetic outflow and increases parasympathetic nerve activity and subsequently improves reflex control of blood pressure. Overall, this reduces blood pressure and protects from the development of hypertension. However, as estradiol levels drop post menopause, baroreflex sensitivity is reduced leading to impaired reflex regulation of blood pressure and subsequently the development of hypertension. Thus, estradiol may interact with stretch sensors within aortic baroreceptors mediates sex-specific differences in the development of hypertension. Figure was generated using BioRender.com.

Estradiol is a steroid hormone that mediates its biological and physiological effects by binding to estrogen receptors (Murdoch and Gorski, 1991). Two forms of estrogen receptors (ER) have been identified, the long-established classical estrogen receptor α (ERα) and the later cloned and discovered estrogen receptor β (ERβ) (Kuiper et al., 1996; Mosselman et al., 1996; Kuiper et al., 1997; Tremblay et al., 1997). Both receptor subtypes have similar binding affinities for estradiol (Mosselman et al., 1996; Kuiper et al., 1997). However, the distribution and expression of both receptors vary by tissue type (Mosselman et al., 1996; Kuiper et al., 1997; Saunders et al., 1997). Both ERα and ERβ are expressed by neurons within the brain and peripheral tissues (Shughrue et al., 1997, 1998; Manolagas and Kousteni, 2001). Interestingly, mRNAs for estrogen receptors are expressed in peripheral sensory neurons (Patisaul et al., 1999; Papka et al., 2001). This includes sensory neurons of the dorsal root ganglia (Papka and Storey-Workley, 2002), neurons within peripheral sympathetic and parasympathetic ganglia (Papka et al., 2001), and within neurons of the nodose ganglion of the vagus nerve (Papka et al., 1997). However, previous studies indicate that ERα not ERβ mediates estrogen facilitation of the baroreflex (Pamidimukkala et al., 2005). For example, *Pamidimukkala* et al., assessed baroreflex sensitivity by challenging the baroreflex with phenylephrine, angiotensin-II and sodium nitroprusside in intact and ovariectomized ERα knockout mice with or without estradiol replacement. They found that baroreflex evoked reductions in HR are blunted in ovariectomized mice and in ERα knockout mice compared to intact control mice. Fascinatingly, estrogen supplement enhanced baroreflex induced bradycardia in ovariectomized mice but not in ERα knockout mice (Pamidimukkala et al., 2005). This study indicates that estradiol enhances BRS through actions on ERα. As ERα are expressed by peripheral sensory neurons, including within the nodose ganglion (Papka et al., 1997, 2001; Papka and Storey-Workley, 2002), the question of whether ERα are expressed by aortic baroreceptors and whether ERα interact with stretch sensor to modulate BRS is raised. While these mechanisms remain to be characterized, similar mechanisms were identified for nociception. For example, studies have demonstrated an interaction between ERα and TRPV1 channels expressed by sensory neurons within the dorsal root ganglion (Cho and Chaban, 2012; Payrits et al., 2017). Estradiol actions on ERα modulate TRPV1 activity which results in modulating nociception and mediating sex differences in pain sensation (Cho and Chaban, 2012; Payrits et al., 2017). Therefore, one can postulate that the actions of estradiol on ERα potentially expressed by aortic baroreceptors enhances the activity of stretch sensors, such as *Piezo*, TRP, and ENaC channels, resulting in enhanced BRS and reflex regulation of BP that subsequently leads to cardioprotective and BP lowering effects in women (Figure 1). Investigating such mechanisms may shed light on the sex-specific differences that mediate reflex regulation of BP and the development of hypertension.

### 4.4 Estradiol and stretch sensors

Although there are no studies examining the actions of estradiol in modulating stretch sensors in aortic baroreceptors, there is extensive evidence that estradiol modulates stretch sensors in other tissues. The example above demonstrates an interaction between estradiol and TRPV1 channels expressed by sensory neurons of the dorsal root ganglion in modulating pain sensation (Cho and Chaban, 2012; Payrits et al., 2017). Similarly, in sensory neurons, estradiol has been shown to enhance the activity of ASIC channels mediating sex-specific differences in acidosis induced nociception (Qu et al., 2015). Similar protective interactions between estradiol and stretch sensors are noted in other tissues. For example, estradiol has been demonstrated to interact with *Piezo2* channels expressed by skeletal muscles to modulate skeletal muscle fibrosis (Potluri et al., 2024). Notably, estradiol actions on ERα expressed by skeletal muscle upregulates *Piezo2* channels which reverses skeletal muscle fibrosis (Potluri et al., 2024). Similarly, the actions of estradiol have been shown to attenuate mechanical stress induced apoptosis of chondrocytes via suppression of *Piezo1* channels in osteoporosis (Sun et al., 2021). Similar interactions between estradiol and aquaporin channels (Cheema et al., 2015; Chinigarzadeh et al., 2016), ENaC channels (Kienitz et al., 2009; Chinigarzadeh et al., 2015), TRPA1 and TRPV1 channels (Pohóczky et al., 2016) are observed in other tissues. In fact estradiol modulates the activity of *Piezo1*, TRPA1 and TRPV1 channels in uterine tissue (Pohóczky et al., 2016; Arishe et al., 2020). Notably, the interaction between estradiol and *Piezo1* channels have been implicated in uterine blood flow during pregnancy and is associated with preeclampsia (Arishe et al., 2020). Thus, there is a body of evidence to suggest a protective interaction between the actions of estradiol on ERα that engage and modulate stretch sensors such as; *Piezo*, TRP, ASIC AQP and ENaC channels. However, while there are no studies to confirm or refute such interactions between estradiol and stretch sensors in aortic baroreceptors, given the plethora of evidence discussed in section 3.2 demonstrating the involvement of stretch sensors in modulating the activity of aortic baroreceptors, and the evidence discussed in section 4.2 demonstrating the protective role of circulating estradiol on BRS, it is reasonable to speculate that similar interaction between estradiol and stretch sensors may be at play in enhancing BRS by engaging aortic baroreceptors (Figure 1B).

### 4.5 Hormone replacement therapy

Thus far, we have discussed the correlation between circulating estradiol and BRS in women (Tanaka et al., 2003). Previous studies demonstrate a link between impaired BRS and the incidence of hypertension (Bristow et al., 1969; Kardos et al., 2001; Indumathy et al., 2015). In fact, the incidence of hypertension is lower in pre-menopausal women when compared to men of similar age, however, the incidence of hypertension increases in post-menopausal women to an equal incidence as in men of similar age (Fryar et al., 2024). Thus, raising the question of whether BRS is different between pre- and post-menopausal women. Indeed, BRS decreases during post menopause and is associated with the increased incidence of hypertension (Subhashri et al., 2021). Interestingly, estrogen replacement therapy improves BRS in post-menopausal women (Hunt et al., 2001). This raises the question of whether estrogen replacement therapy reduces the risk of hypertension and cardiovascular disease in post-menopausal women. Hormone replacement therapy in post-menopause has been explored in the Women's Health Initiative Randomized Trials and Clinical Practice between 1990–2000 (Manson et al., 2024). Although the trial demonstrated that estradiol replacement in post-menopausal women does not prevent cardiovascular disease (Manson et al., 2024), subsequent age-stratified analyses with longer cumulative follow-up supported a more nuanced approach to hormone replacement therapy (Prentice and Anderson, 2008; Cho et al., 2023). In that, incidence of cardiovascular disease was lower when estradiol replacement was initiated in younger peri-menopause women (50–59 years of age) as opposed to older women (a decade after menopause) (Prentice and Anderson, 2008). A plethora of follow up clinical studies demonstrated that estradiol supplement in peri-menopausal women (45–55 years of age) reduces the incidence of cardiovascular disease and improves vascular health in post-menopausal ages (Wenner et al.; Moreau et al., 2002, 2013). Thus, indicating the importance of timing of initiating estradiol replacement therapy in reducing the incidence of cardiovascular disease. Importantly, these studies suggest a protective role circulating estradiol plays ion enhances BRS and reducing the incidence of hypertension and cardiovascular disease. While a plethora of studies investigate the role of estradiol in enhancing BRS leading to a reduced incidence of cardiovascular disease, fewer studies investigate the role of other sex hormones in cardiovascular disease. Studies in human subjects indicate that progesterone opposes the actions of estradiol on BRS. In that the administration of progesterone blunts BRS in young women, however, estradiol administration enhances BRS (Brunt et al., 2013). This is in agreement with the studies mentioned above demonstration that baroreflex sensitivity is at its highest during the pre-ovulation phase when estradiol levels are peaking and progesterone levels are low (Tanaka et al., 2003). Similarly, studies indicate a correlation between testosterone levels and BRS, where low testosterone levels in men correlates to reduced BRS and high incidence of cardiovascular disease (El-Mas et al., 2001; Rydlewska et al., 2013). Altogether, these studies link a reduced BRS to an increases incidence of cardiovascular disease in post-menopause, which is attenuated with estradiol suplementation.

## 5 Discussion and future directions

The role the baroreflex plays in the development of hypertension is an evolving area of research. In the current review, we addressed fundamental concepts and current knowledge gaps in the field of aortic baroreceptors. Aortic baroreceptors are sensory neurons that innervate the aortic arch (Cheng et al., 1997; Min et al., 2019; Elsaafien et al., 2022). These sensory neurons can sense changes in blood pressure by sensing vascular stretch at the aortic arch (Baumer-Harrison et al., 2024). Thus, aortic baroreceptors employ ion channels, or stretch sensors, to detect vascular stretch. Stretch sensors are activated as the cellular membrane is stretched, allowing for calcium ions to flow into the cell and subsequently depolarizing the cell (Gomis et al., 2008; Dong et al., 2010; Ranade et al., 2014; Woo et al., 2014). Once aortic baroreceptors are depolarized, action potentials are conveyed through the aortic depressor nerve which connects into the vagus nerve, where these signals are then transmitted to the hindbrain by the vagus nerve (Cottle, 1964; Lipski et al., 1975; Mendelowitz et al., 1992; Elsaafien et al., 2022; Scott et al., 2025). Once the signal reaches the nucleus of the solitary tract in the hindbrain, autonomic outflow is modulated to regulate blood pressure at homeostatic levels (Ismay et al., 1979; Lee et al., 1980; Guo and Abboud, 1984) (Figure 1). Here, we questioned the identity of ion channels implicated in sensing vascular stretch that are employed by aortic baroreceptors. Although the mechanically gated ion channels *Piezo1* and *Piezo2* receive the spotlight in the field of stretch sensation (Zeng et al., 2018; Min et al., 2019), many ion channels are implicated in sensing vascular stretch in aortic baroreceptors (Yang et al., 2022). We highlighted several transient receptor potential channels, acid sensing ion channel, and epithelial sodium channels that are expressed and engaged by aortic baroreceptors to evoke the baroreflex. This concept may raise the concern that the stretch sensors employed by aortic baroreflex are redundant. Aortic baroreceptors signal to the hindbrain through vagal afferent that are either myelinated (A-fibers) or unmyelinated (C-fibers) (Seagard et al., 1993; Fan et al., 1996; Fan and Andresen, 1998; Armstrong and Moore, 2023). Myelinated A-fibers are utilized in dynamic transmission and are involved in transient changes of blood pressure (Seagard et al., 1993). Whereas, unmyelinated C-fibers are engaged in tonic transmission to evoke sustained changes in blood pressure (Fan and Andresen, 1998). *In vivo* intravital calcium imaging of aortic baroreceptors revealed two distinct responses following a pressor challenge: early and late responder aortic baroreceptors (Baumer-Harrison et al., 2024). Early responders increase their activity immediately, and lasting for 60s, following a pressor challenge. Late responders increase their activity 60–80 s after a pressor challenge and remain active for the duration of the recording. Under physiological conditions, most aortic baroreceptors are early responders whereas in a mouse model of hypertension the majority become late responders (Baumer-Harrison et al., 2024). Once can postulate that early responders utilize A-fibers in dynamic and transient signaling to regulate blood pressure, whereas late responders utilize C-fibers in sustained changes of blood pressure. Thus, raising the question of whether stretch sensors are differentially expressed by the different aortic baroreceptive fibers. Indeed, while most stretch sensors are expressed by both A and C fibers, their relative expression between fibers is differential (Longhurst et al., 1984; Glazebrook et al., 2005; Szczot et al., 2021; Lee et al., 2025). For example, *Piezo* channels are predominantly expressed in A-fibers (Cui et al., 2024), whereas TRPV1 channels are mainly expressed by C-fibers (Mohammed et al., 2018). This may explain that while the knockout of *Piezo* channels from aortic baroreceptors impairs baroreflex sensitivity, it does not lead to the development of a hypertensive phenotype but rather a blood pressure labile phenotype (Zeng et al., 2018; Min et al., 2019). In addition, it indicates that stretch sensors may be differentially involved in blood pressure regulation by signaling differentially through A and C fibers. Furthermore, some stretch sensors may be involved in the initial detection of vascular stretch, while others may be involved in maintaining the stretch sensation. This would require interaction between the different stretch sensors. Several studies highlight an interaction between the different ion channels to evoke stretch-induced changes in blood pressure (Galizia et al., 2012; Yan et al., 2021; Lu et al., 2022). For example, in vascular smooth muscle cells both epithelial sodium and acid sensing ion channels are required to elicit pressure-induced vasoconstriction in renal afferent arterioles (Lu et al., 2022). Intriguingly, AQP4 and TRPV4 channels were found to interact and be required for evoking responses to cellular swelling following a hypotonic challenge (Benfenati et al., 2011). AQP4 channels transport water across the cell to reduce cellular volume in response to cellular swelling. TRPV4 channels are activated following a hypotonic challenge, where calcium flows into the cell and subsequently activates AQP4 channels. Knockout of both AQP4 and TRPV4 channels from astrocytes impairs the ability of astrocytes to detect or regulate cellular volume in response to cellular swelling (Benfenati et al., 2011). Deletion of only TRPV4 channels abolishes the cell's ability to detect cellular swelling. Whereas deleting AQP4 channels impairs cellular volume regulation but does not stop calcium influx into the cell in response to hypotonic stress (Benfenati et al., 2011). This offers an example for two different ion channels interacting, where one channel detects cellular swelling and the other evokes a response to it. In summary, baroreception is a complex physiological reflex that involves different fibers and stretch sensors to evoke, prime, maintain and fine tune the reflex to achieve blood pressure homeostasis under many different physiological conditions. Dysfunction or impairment of this complex feedback mechanism can lead to the development of hypertension and cardiovascular disease.

The involvement of the baroreflex in the development of hypertension is debatable. Earlier studies suggest that the link between baroreflex impairment and hypertension to be questionable. Ablation of sensory neurons innervating the carotid sinus and the aortic arch were not found to induce a hypertensive phenotype (Norman et al., 1981; Buchholz et al., 1986; Barron et al., 1989; Schreihofer and Sved, 1992). Thus, giving rise to the common interpretation that the baroreflex is not implicated in the development of hypertension. Interpretations of these studies are challenging. Firstly, previous studies have shown that while the carotid sinus contains arterial baroreceptors, aortic baroreceptors exhibit higher mechano-sensitivity than carotid baroreceptors (Lau E. O. C. et al., 2016). Furthermore, the carotid sinus contains arterial chemoreceptors which oppose the effects of arterial baroreceptors (Seagard et al., 1990; Silva et al., 2015; Porzionato et al., 2019). Hence, ablating sensory neurons at both the carotid sinus and the aortic arch makes it challenging to draw any conclusions for the role arterial baroreceptors play in the development of hypertension. In fact, a plethora of previous studies have shown impaired baroreflex sensitivity in rodent models of hypertension (Ismay et al., 1979; Lee et al., 1980; Guo and Abboud, 1984; Bishop and Sanderford, 2000; Sanderford and Bishop, 2000, 2002). Intriguingly, reduced baroreflex sensitivity precedes sympathetic overactivity which occurs prior to sustained elevations in blood pressure and the development of hypertension (Oliveira-Sales et al., 2014, 2016). These studies highlight an important role the baroreflex plays in the development of hypertension. In fact, in the clinical setting, reduced baroreflex sensitivity is associated with hypertension (Bristow et al., 1969; Kardos et al., 2001; Indumathy et al., 2015). Intriguingly, women exhibit enhanced baroreflex sensitivity compared to men (Tanaka et al., 2003; Man et al., 2021). This may explain the incidence and the severity of hypertension being lower amongst women than men (Kotchen et al., 1982; Schenck-Gustafsson, 1996; Dubey et al., 2002). Fascinatingly, baroreflex sensitivity is both correlated and associated to systemic estradiol levels. During the pre-ovulation phase where estradiol levels peak, women have an enhanced baroreflex sensitivity which is reduced post-ovulation, where estradiol levels drop (Tanaka et al., 2003). This indicates that estradiol can modulate baroreflex sensitivity to regulate reflex control of blood pressure. In estradiol-low state, such as in men or during post-ovulation and post-menopause in women, baroreflex sensitivity is reduced leading to elevated blood pressure. Mechanistically, estradiol has been shown to modulate the activity of stretch sensors in other tissues (Cho and Chaban, 2012; Payrits et al., 2017). For example, estradiol regulates the expression of TRPA1 and TRPV1 in the endometrium (Pohóczky et al., 2016). Furthermore, it regulates the expression of TRPV4 channels in the hindbrain of spontaneously hypertensive rats (Onishi et al., 2018), suggesting an association between estradiol and TRPV4 channels in mediating sex-specific differences in hypertension. Here we propose that estradiol modulates the activity of stretch sensors in aortic baroreceptors leading to enhanced baroreflex sensitivity (Figures 1A, B). This subsequently leads to enhanced autonomic reflex regulation of parasympathetic and sympathetic outflow (Figure 1C). We propose that these mechanisms may be implicated in mediating sex-specific differences in hypertension, where both the incidence and the severity of hypertension are lower amongst pre-menopausal women than men and post-menopausal women (Figure 1).

## 6 Conclusion

In conclusion, the current review highlights aortic baroreceptors as key modulators of blood pressure, where dysfunction can lead to the development of hypertension. We propose that several of ion channels to be involved in vascular stretch sensation employed by aortic baroreceptors. This may include *Piezo*, transient receptor potential, acid sensing ion, epithelial sodium, and aquaporin channels. Furthermore, these ion channels are differentially expressed by the different myelinated and unmyelinated aortic baroreceptors fibers and may be involved in detecting, maintaining and regulating the stretch signal differentially. Finally, we propose that stretch sensors employed by aortic baroreceptors can be modulated by circulating estradiol levels and thus mediate sex-specific differences in hypertension.
